# Book Notices

**Published:** 1881-10-20

**Authors:** 


### *BOOK NOTICES.*

A TREATISE ON DISEASES OF THE JOINTS—By Richard Barwell, F. R. C. S., Senior Surgeon and Lecturer on Surgery, Charing Cross Hospital—Illustrated by numerous engravings on wood. Second edition, revised and much enlarged. New York, William Wood & Co., 1881; W. B. Dalston, Agent, Atlanta, Ga.

A book of 457 octavo pages. Among the important subjects discussed are acute synovitis, pyæmic joint disease, strumous synovitis, rheumatic synovitis and syphilitic and gouty synovitis, hip-joint disease, ostitis, restoration of crippled joints, genu varum and valgum, etc. A list of valuable formulæ is found at the close of the work. We regared the work as eminently practical and instructive.

TRANSACTIONS OF THE COLLEGE OF PHYSICIANS AND SURGEONS of Philadelphia, Penn., Volume Twelfth. Printed for the College and for sale, by Lindsay & Blakiston, 1881.

Subjects treated: Memoirs of George B. Wood, Dr. Isaac Hays, Dr. John Marshall Paul, Dr. Jas. Aitkin Meigs, Dr. Thaddeus L. Leavitt, Dr. John Neill, Dr. Isaac Ray.

Foot-binding in China, etc., by Dr. R. P. Harris, A. M., M. D.

Account of a case of Heart Clot, etc., by Arthur V. Meigs, M. D., and remarks on by J. B. Roberts, M. D.

Report of the Com. on Meteorology, by Rich'd A. Cleeman, M. D.

Case of General Hyperostosis, by Jos. H. Hutchinson, M. D.

Case of Starvation Fever, by J. M. Da Costa, M. D.

Report of a case of Diabetes Mellitus in which double Cataract existed, by J. E. Mears, M. D.

Thoughts upon Vivisection, etc., by George Hamilton, M. D., and remarks on, by J. C. Morris, M. D.

A SYSTEM OF SURGERY, THEORETICAL AND PRACTICAL, IN TREATISES—By various authors, edited by T. Holmes, Cantab, Surgeon and Lecturer on Surgery at St. George's Hospital. Memb. Correp. De La Societe De Chirurgie De Paris. First American from second English edition, thoroughly revised and much enlarged, by John H. Packard, A. M., M. D., Surgeon to the Episcopal and St. Joseph's Hospitals, Philadelphia, assisted by a large corps of the most eminent American Surgeons, in three volumes, with many illustrations. Vol. 1—General Pathology; Morbid Processes; Injuries in General; Complications in Injuries; Injuries of Regions. Philadelphia, Henry C. Lea's Son & Co.

Dr. Packard, in the preface to the American edition, justly remarks that "The high position universally accorded to Holmes' system of Surgery has rendered it very desirable that this work, containing in in itself a vast store of the learning and experience of some of Great Britain's best surgeons, should be made more available to the profession in this country; and that the new material, accumulated on both sides of the Atlantic, in the ten years which have elapsed since its publication, should be incorporated in it."

In the volume before us, being the first of three that are to be issued, we have a neat and elegant work of over 1,000 pages, printed in double column, and beautifully illustrated, containing an able and systematic treatment of general surgery and injuries of regions. In the forthcoming volumes we are to have the "Diseases of the various systems and organs, and the concluding portion will be devoted to general practical matters—operative and minor surgery, gunshot wounds, hospitals" and other topics. There is to be a copious index at the end of each volume "and a general one at the conclusion of the whole." This work should be in the library of every physician and surgeon.

COULSON ON DISEASES OF THE BLADDER AND PROSTATE GLAND, sixth edition, revised by Walter J. Coulson, F. R. C. S., Surgeon to St. Peter's Hospital for Stone, etc., and Surgeon to the Sack Hospital; New York, William Wood & Co., 27 Great Jones Street, 1881. Oct. pp. 393.

From a careful examination of the above work we are convinced of its value to the practitioner embracing as it does a more full and complete treatise on the subject than any recent work with which we are acquainted. The book is illustrated and contains a very full index, making it a convenient work of reference to the busy practitioner.

